# Model compression for real-time object detection using rigorous gradation pruning

**DOI:** 10.1016/j.isci.2024.111618

**Published:** 2024-12-17

**Authors:** Defu Yang, Mahmud Iwan Solihin, Yawen Zhao, Bingyu Cai, Chaoran Chen, Andika Aji Wijaya, Chun Kit Ang, Wei Hong Lim

**Affiliations:** 1 Faculty of Engineering, Technology and Built Environment, UCSI University, Kuala Lumpur, Malaysia; 2 School of Advanced Manufacturing, Shantou Polytechnic, Shantou, China; 3 Department of Mechanical Engineering, University of Business & Technology, Jeddah, Saudi Arabia

**Keywords:** Artificial intelligence, Engineering

## Abstract

Achieving lightweight real-time object detection necessitates balancing model compression with detection accuracy, a difficulty exacerbated by low redundancy and uneven contributions from convolutional layers. As an alternative to traditional methods, we propose Rigorous Gradation Pruning (RGP), which uses a desensitized first-order Taylor approximation to assess filter importance, enabling precise pruning of redundant kernels. This approach includes the iterative reassessment of layer significance to protect essential layers, ensuring effective detection performance. We applied RGP to YOLOv8 detectors and tested it on GTSDB, Seaships, and COCO datasets. On GTSDB, RGP achieved 80% compression of YOLOv8n with only a 0.11% drop in mAP0.5, while increasing frames per second (FPS) by 43.84%. For YOLOv8x, RGP achieved 90% compression, a 1.26% mAP0.5:0.95 increase, and a 112.66% FPS boost. Significant compression was also achieved on Seaships and COCO datasets, demonstrating RGP’s robustness across diverse object detection tasks and its potential for advancing efficient, high-speed detection models.

## Highlights

• A desensitized Taylor approximator for filter significance in object detection networks
• Gradient-based pruning with the reevaluation of each layer’s importance, layer-by-layer
• Rigorous gradient pruning enables significant YOLOv8 compression while preserving accuracy
• Object detection model efficacy is evaluated using the GTSDB, Seaships, and COCO datasets

## Graphical abstract

**Figure:**
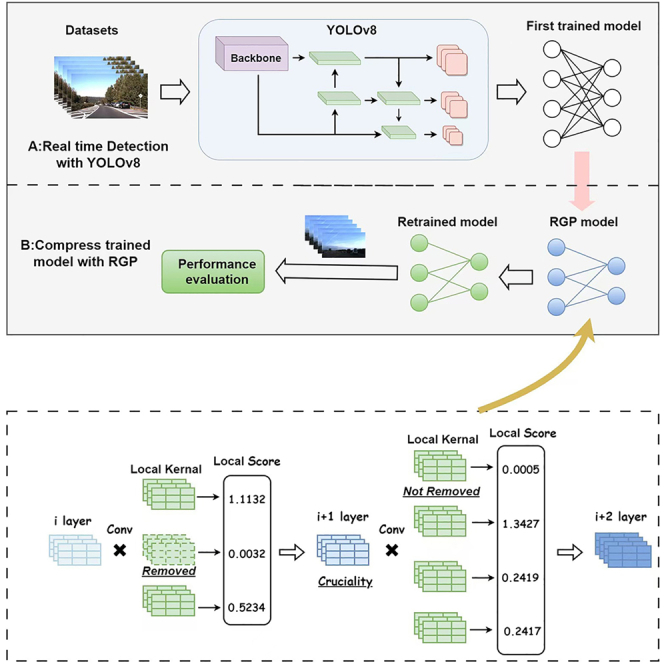

## Introduction

Object detection based on machine vision holds substantial potential due to the detailed information provided by the visual sensors it relies on, which also offer significant cost advantages.^1^ In recent years, object detection models have increasingly been applied across various domains, such as quality control in manufacturing,^2^ automotive,^3^ and ship tracking and detection.^4^ Concurrently, numerous real-time network architectures have been introduced to meet the demands of real-time processing. Among these, the YOLO (You Only Look Once) series is particularly renowned for its high detection accuracy and real-time performance.^5^

Real-time object detection networks, such as YOLO, rely on deep convolutional layers to extract and process semantic information. While these layers improve detection accuracy, they also greatly increase computational demand and parameters, creating challenges in real-time applications where speed and energy efficiency are critical. Expanding the depth or width of convolutional layers can boost accuracy but also add latency, which is problematic for time-sensitive tasks such as autonomous driving, illustrating the trade-off between accuracy and speed. The high computational demands of YOLO models also lead to increased energy consumption and deployment costs, particularly in resource-constrained environments such as edge computing and mobile devices. Although measures have been taken to reduce network redundancy, these efforts may still not fully eliminate the impact of complexity on real-time performance, making further network compression essential.

### Compressing network structures while maintaining detection accuracy presents a formidable challenge

Detection accuracy is typically associated with the depth and complexity of a network. Traditional methods such as MobileNet^6^ and NASNet,^7^ which adjust network structures for lightweighting, often have limited impact on real-time network architectures. This is particularly evident in advanced versions of the YOLO series, where many redundancies have been addressed, and compression frequently results in diminished detection performance. Furthermore, traditional pruning methods encounter difficulties because real-time network architectures place greater emphasis on the output layers, and the importance scores of convolution kernels are more concentrated, making it easy to degrade performance without a meticulous pruning strategy inadvertently.

**Figure 1:**
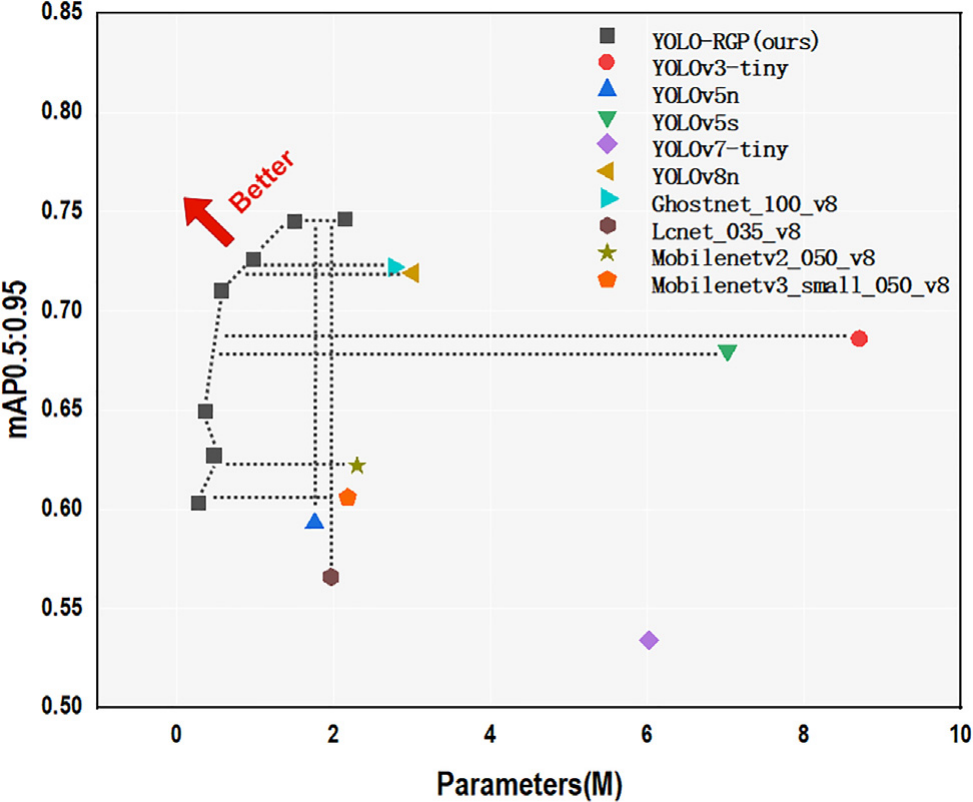
Performance Comparison of Our Proposed YOLO-RGP Model with Mainstream Models YOLO-RGP represents the newly compressed model obtained by applying our proposed RGP method to YOLOv8n. In this figure, we have selected seven versions of YOLO-RGP, ordered from smallest to largest based on their parameter sizes. The corresponding speed-up factors are 10, 8, 6, 5, 3, 2, and 1.4, respectively.

To address these issues, we developed a lightweight RGP model that prunes real-time network structures based on the importance scores of filters within each layer, systematically removing unimportant filters to streamline the network. We implemented our RGP method in YOLOv8^8^ and evaluated it on several datasets, including GTSDB,^9^ Seaships,^10^ and MS COCO 2017,^11^ achieving satisfactory compression results without compromising detection accuracy. Figure 1 illustrates the performance of our pruned network structure, YOLO-RGP, in comparison with other mainstream object detection networks, demonstrating that our model consistently outperforms the others. YOLO-RGP represents the newly compressed model obtained by applying our proposed RGP method to YOLOv8n. As shown in Figure 1, we have selected seven versions of YOLO-RGP, ordered from smallest to largest based on their parameter sizes. The corresponding speed-up factors are 10, 8, 6, 5, 3, 2, and 1.4, respectively.

Our main contributions are as follows:

(1) We propose a desensitized first-order Taylor approximator to reassess the importance of filters within network architectures. By integrating an exponent into the traditional first-order Taylor expansion, our method mitigates issues associated with slope sensitivity, thereby yielding distinguishable scores even in scenarios where filter scores are highly concentrated.
(2) We propose a highly effective RGP for model compression. This approach assesses each layer’s contribution to the overall output and methodically prunes filters by layer based on their importance scores, derived by aggregating scores from more fine-grained elements. This strategy not only offers good universality but also ensures more precise pruning effects.
(3) We propose a strategy for successfully deploying the RGP into YOLOv8, with a special consideration of the importance of each layer in the real-time object detection network, particularly those related to classification and localization. Experimental results demonstrate that our compressed real-time network structure maintains detection accuracy while requiring fewer parameters and computational costs.

### Object detection models

Traditional object detection models rely on prior knowledge for tasks such as identifying regions of interest,^12^ extracting features,^13^ and classifying and localizing objects.^14^ These models often face limitations in robustness and scalability due to their dependence on predefined rules and manual feature extraction, which can hinder their performance in diverse and complex scenarios. In contrast, CNN-based deep learning models address some of these issues through one-stage and two-stage approaches. Two-stage models, such as R-CNN (Region-based Convolutional Network),^15^ Fast R-CNN,^16^ Faster R-CNN,^17^ and Mask R-CNN,^18^ offer improved accuracy but often struggle with real-time performance due to their computational complexity and slower processing speeds. One-stage models, including SSD (Single Shot MultiBox Detector),^19^ RetinaNet,^20^ CornerNet,^21^ CenterNet,^22^ and YOLO,^5^ enhance efficiency by eliminating the candidate box phase, which improves real-time performance, with the YOLO series receiving significant attention. However, YOLO models, particularly those with deep architectures, still face significant challenges related to high computational demands and parameter counts. These challenges can limit their scalability and deployment in resource-constrained environments, highlighting the ongoing need for effective network compression to optimize accuracy, speed, and resource efficiency.

### Compression methods

Regarding model compression, optimization is typically achieved by modifying the network structure or by applying pruning methods to eliminate certain components. Adjusting the network structure is a widely adopted approach. For instance, MobileNet^6^ and Xception^23^ use Depthwise Separable Convolutions to replace traditional convolutions, reducing the parameter count by decomposing standard convolutions into channel-wise and pointwise operations. NAS is another prominent method, as seen in models such as NASNet^7^ and MobileNetv3,^24^ which utilize automatic searches to identify efficient network architectures. MnasNet^25^ extends this by directly incorporating model latency into the optimization process, striking a balance between accuracy and latency. However, NAS methods often demand significant training time, posing a notable limitation. Group Convolution, as demonstrated by models such as ShuffleNet^26^ and ResNeXt,^27^ reorganizes channels into groups, thus enhancing the flow of information within these groups and reducing computational complexity. In addition, models such as GhostNet^28^ offer more computationally efficient operations to replace standard convolutions. Similarly, LCNet^29^ adopts this strategy, employing Ghost Modules for compression while using dilated convolutions to expand the receptive field. The Inception^30^ modules also play a significant role in enhancing the efficiency of feature extraction. However, despite the increasing efficiency of advanced real-time object detection networks, merely adjusting the network structure often proves insufficient for significant model compression. Such structural modifications can result in a substantial drop in detection accuracy when applied without more nuanced methods.

Another compressive approach is pruning, which can be broadly categorized into unstructured, dynamic, and structured types. Unstructured pruning typically involves removing individual weights, which can result in high sparsity and often requires specialized libraries to manage sparse matrices. This method may impact detection accuracy due to the introduction of excessive sparsity, making it challenging to maintain model performance. Dynamic pruning, as demonstrated by CHEX^31^ and DDG,^32^ adjusts pruning decisions continuously based on real-time network performance metrics. While this approach is more adaptive, it comes with higher computational costs due to the need for ongoing performance monitoring and adjustments.

In contrast, structured pruning, which focuses on removing entire filters or channels rather than individual weights, has garnered significant attention from researchers. Structured pruning involves identifying and eliminating less critical parts of the network based on their importance, which simplifies the network architecture and often enhances computational efficiency. For example, DHP^33^ and MetaPruning^34^ leverage NAS to automatically identify and prune non-essential structures; however, this approach demands substantial search time. Additionally, methods such as APoZ^35^ and PFP^36^ assign values to neurons and discard those with values close to zero, indicating failed activations. While straightforward, this activation-based approach is computationally demanding due to the multiple iterations required to verify activation values. Techniques such as LeGR^37^ and OTO^38^ employ regularization terms to sparsify weight values, targeting weights that diminish to zero. Although effective, these techniques can be challenging to implement as they may lead to suboptimal pruning plans, potentially compromising detection performance. PFEC^39^ emphasizes the importance of filters and systematically eliminates less critical ones to streamline the network. However, accurately ordering filters is crucial, and in deep network structures, the specific contributions of individual layers are often overlooked, which can lead to incorrect deletions and reduced performance.

**Figure 2:**
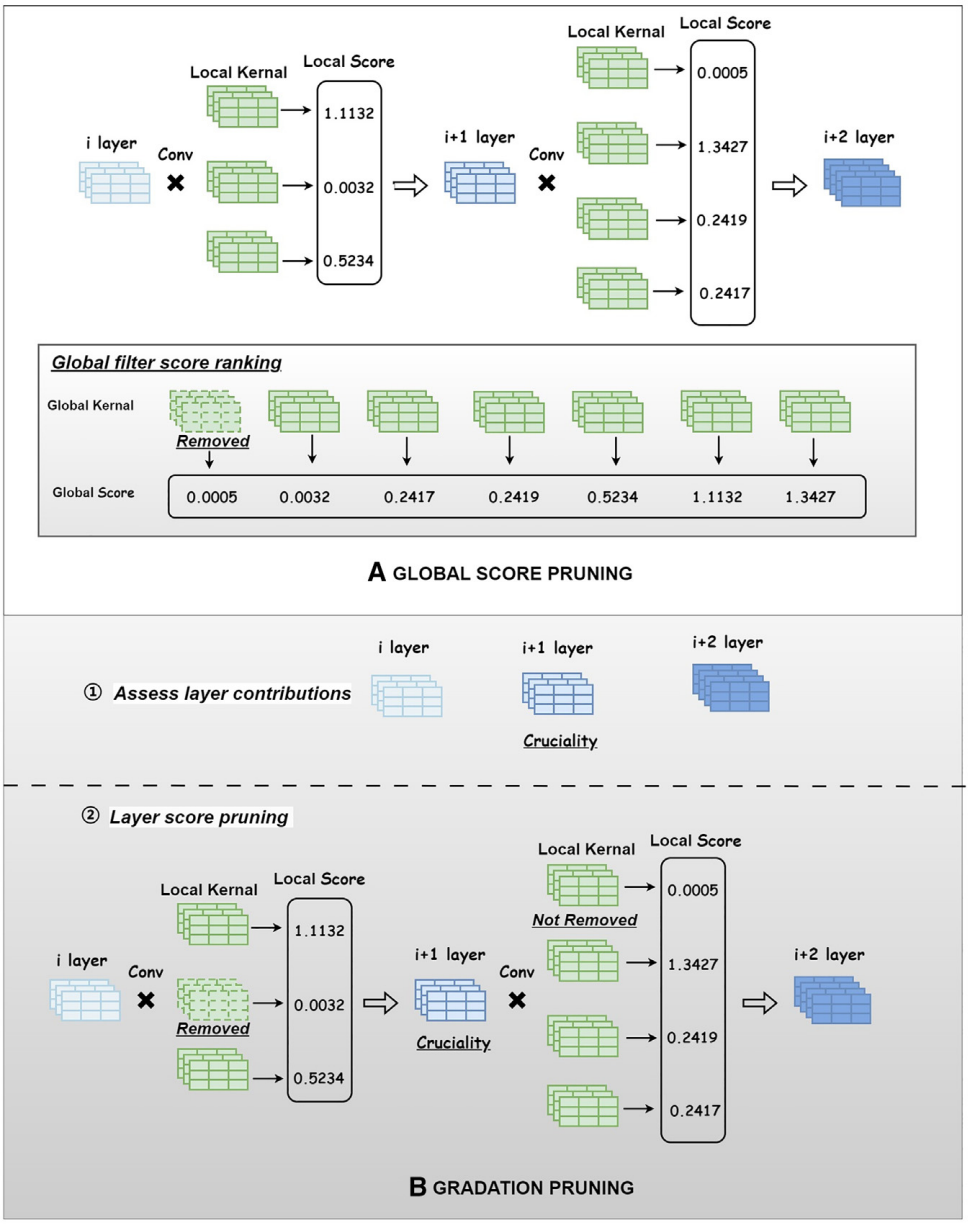
Pruning Process Based on Filter Importance Scores In process A: GLOBAL SCORE PRUNING, the importance scores of filters within each layer are first calculated. Then, the convolution kernels' scores from all layers are ranked globally, and the kernels with the lowest scores are pruned incrementally based on the required pruning rate. In process B: GRADATION PRUNING, the impact of each layer’s structure on the output is initially assessed. Subsequently, the importance scores of the filters within each layer are ranked locally, and the filters with the lowest scores in each layer are pruned. Pruning is avoided in crucial layers.

**Figure 3:**
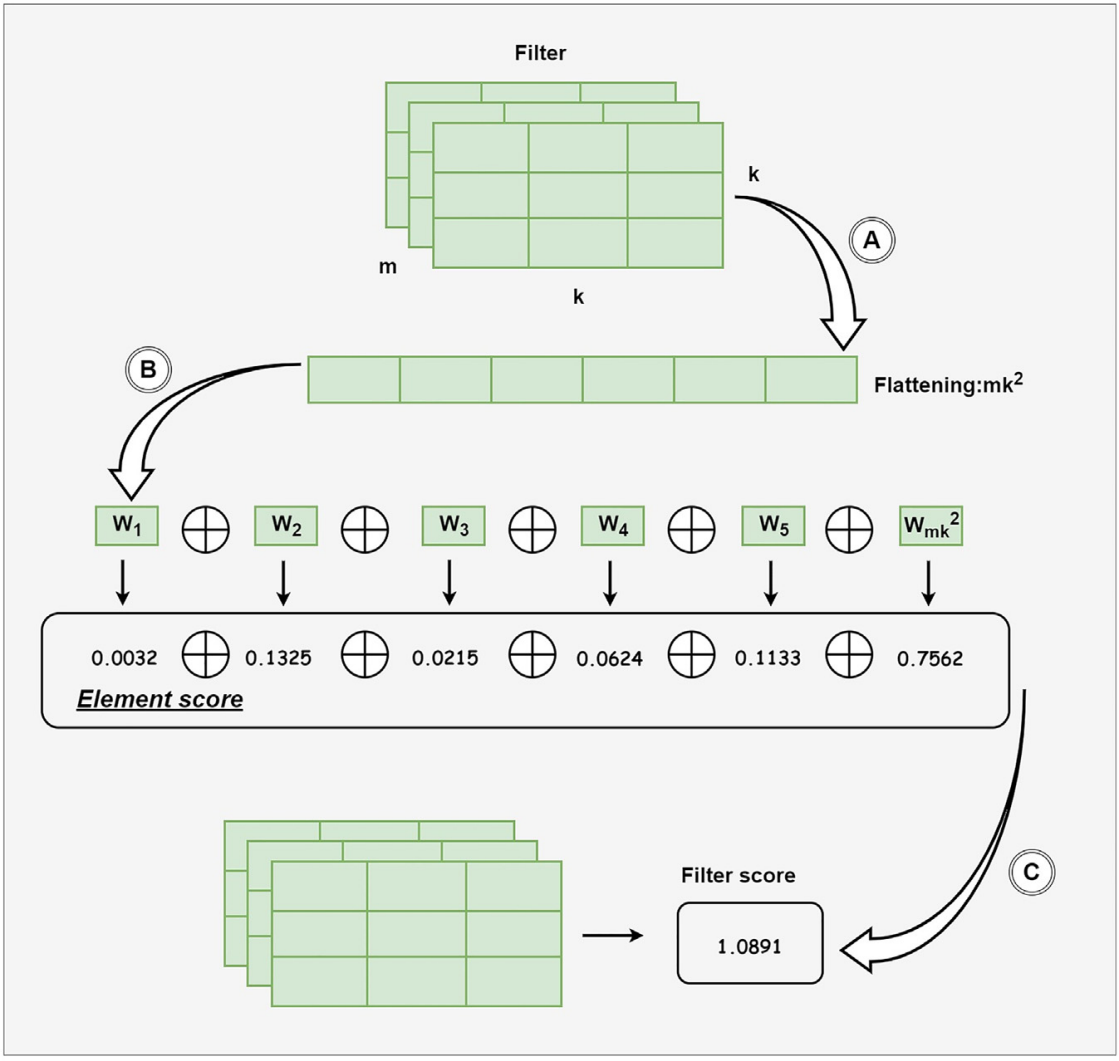
A Fine-Grained Method for Assessing the Importance of Convolution Kernels The convolution kernel itself consists of m input channels, with each channel having a convolution size of k∗k, resulting in a structure of [m, k, k] channels. Operation A involves flattening the multi-dimensional convolution kernel into a one-dimensional array of mk^2^ to facilitate subsequent computations and sorting. In Operation B, the importance scores for each element are calculated. Finally, Operation C combines all the element scores to form the overall filter score.

**Figure 4:**
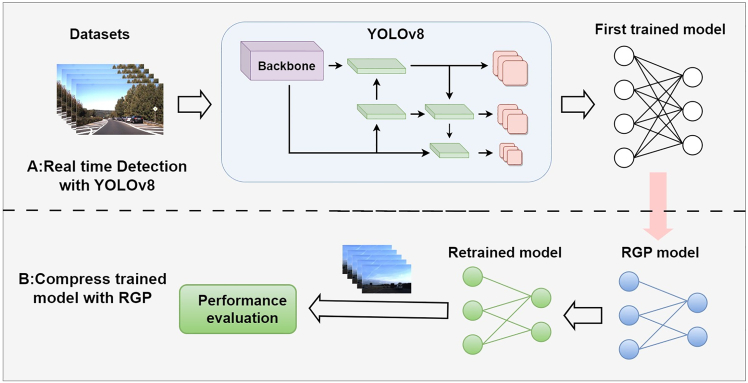
The specific implementation process of RGP in YOLOv8 (A) Realtime Detection with Yolov8. (B) Compress trained model with RGP.

This article, building on traditional structured pruning methods, adopts a compressive approach based on importance scores of filters derived from Taylor expansion.^40^ This approach aims to achieve a more effective compression of real-time object detection models through rigorous graded pruning. See Figures 2, 3, and 4 for the detailed implementation of the pruning process based on filter importance scores.

## Results

### Comparison with mainstream detection models in GTSDB dataset

In this section, we compare our proposed methods with state-of-the-art models on the GTSDB dataset. Our approach includes two variants: YOLO-RGPn and YOLO-RGPs, which are lightweight adaptations of the YOLOv8n model using the RGP method, with speed-ups of 2x and 1.4x, respectively. As shown in Table 1, YOLO-RGPs achieves the highest mAP0.5 and mAP0.5:0.95 scores among several models, reaching 0.937 and 0.747, respectively. This represents an improvement of 1.85% and 3.89% over the baseline model, highlighting a significant boost in detection accuracy after reducing redundant parameters. Our other variant, YOLO-RGPn, also ranks second in both of these metrics.

**Table 1:** Comparison results with mainstream detection models in the GTSDB dataset: the best results are indicated with both bold and underlined, while the second-best results are marked with an underline only

Model	mAP0.5	mAP0.5:0.95	PC(M)	FLOPs(B)	FPS
YOLOv3-tiny^41^	0.930	0.686	8.7	12.9	131.58
YOLOv5n^42^	0.828	0.593	1.8	4.1	**133.33**
YOLOv5s^42^	0.902	0.680	7.0	15.8	129.87
YOLOv6n^43^	0.900	0.708	4.6	11.3	116.28
YOLOv7-tiny^44^	0.752	0.534	6.0	13.0	79.37
YOLOv8n^8^	0.920	0.719	3.0	8.1	95.24
YOLO-RGPn (ours)	0.931	0.745	**1.5**	**4.0**	101.01
YOLO-RGPs (ours)	**0.937**	**0.747**	2.2	5.8	92.59

Additionally, YOLO-RGPn exhibits exceptional performance in lightweight efficiency metrics, with a PC (M) of 1.5 and FLOPs (B) of 4.0. These values reflect a compression of −50.00% and −50.62%, respectively, compared to the baseline model, while maintaining competitive detection accuracy. Moreover, in terms of FPS, although YOLO-RGPn does not take the leading position, it achieves a 6.06% improvement over the baseline model, demonstrating robust real-time performance. These results thoroughly demonstrate the effectiveness of our proposed methods in achieving lightweight and real-time capabilities on the GTSDB dataset.

### Comparison with mainstream detection models in Seaships dataset

To further validate the effectiveness of our proposed method across different scenarios, we conducted experiments using the Seaships maritime dataset. As illustrated in Table 2, in terms of mAP0.5, our YOLO-RGPs model achieved the highest score. For mAP0.5:0.95, YOLO-RGPs and YOLO-RGPn achieved scores of 0.806 and 0.801, respectively, securing the first and second positions, and were the only models among all tested to surpass the 0.8 threshold. Regarding PC (M) and FLOPs (B), YOLO-RGPn consistently maintained its leading position, further underscoring the efficiency of the RGP compression method. In terms of FPS, both YOLO-RGPn and YOLO-RGPs ranked first and second, respectively, once again demonstrating the superiority of our models in real-time performance. These results clearly affirm the effectiveness of our proposed models in maritime environments.

**Table 2:** Comparison results with mainstream detection models in the Seaships dataset: the best results are indicated with both bold and underline, while the second-best results are marked with an underline only

Model	mAP0.5	mAP0.5:0.95	PC(M)	FLOPs(B)	FPS
YOLOv3-tiny^41^	0.981	0.730	8.7	12.9	98.03
YOLOv5n^42^	0.981	0.745	1.8	4.2	90.90
YOLOv5s^42^	0.988	0.780	7.0	15.8	71.94
YOLOv6n^43^	0.989	0.792	4.6	11.3	101.73
YOLOv7-tiny^44^	0.981	0.742	6.0	13.1	97.08
YOLOv8n^8^	0.986	0.783	3.0	8.1	97.08
YOLO-RGPn (ours)	0.984	0.801	**1.5**	**4.0**	**123.46**
YOLO-RGPs (ours)	**0.991**	**0.806**	2.1	5.8	108.70

### Comparison with mainstream detection models in COCO dataset

Furthermore, we compared the performance of our proposed models against other mainstream models on the large COCO dataset, as shown in Table 3. Compared to YOLOv3-tiny, YOLOv4-tiny, and YOLOv5n, our size-equivalent YOLO-RGPn achieved first place in both mAP0.5 and mAP0.5:0.95, despite being slightly behind in FPS. Notably, YOLO-RGPn outperformed the second-best YOLOv5n in mAP0.5:0.95 by 12.36%. Additionally, YOLO-RGPn maintained the smallest parameter count and computational cost in terms of PC (M) and FLOPs (B).

**Table 3:** Comparison results with mainstream detection models in the COCO dataset: the best results are indicated with both bold and underline, while the second-best results are marked with an underline only

Model	mAP0.5	mAP0.5:0.95	PC(M)	FLOPs(B)	FPS
YOLOv3-tiny^41^	0.360	0.179	8.9	12.9	**172.41**
YOLOv4-tiny^44^	0.421	0.249	6.1	6.9	–
YOLOv5n^42^	0.449	0.275	1.9	4.5	112.36
YOLOv5s^42^	**0.545**	0.361	7.3	16.5	113.64
YOLOv6n^43^	0.521	**0.369**	4.7	11.4	87.11
YOLOv7-tiny^44^	0.528	0.352	6.2	5.8	–
YOLOv8n^8^	0.519	**0.369**	3.2	8.7	116.28
YOLO-RGPn (ours)	0.450	0.309	**1.6**	**4.3**	111.11
YOLO-RGPs (ours)	0.486	0.339	2.2	6.2	105.26

When compared to YOLOv5s, YOLOv6, YOLOv7-tiny, and YOLOv8n, our recommended YOLO-RGPs may slightly lag in accuracy performance but offers substantial improvements in lightweight efficiency. For instance, compared to YOLOv5s, although YOLO-RGPs sacrifices 10.83% and 6.09% in mAP0.5 and mAP0.5:0.95, respectively, it achieves a remarkable 69.86% reduction in PC (M) and 62.42% in FLOPs (B), making it a more attractive option overall. These results further demonstrate the viability of our proposed methods.

### Comparison with some compression models

To further validate the advantages of our proposed method, we used YOLOv8n as the baseline model and applied different compression techniques for improvement, where RGP-1.4 and RGP-2 represent the acceleration of the baseline model by 1.4x and 2x, respectively, using the RGP method. As shown in Table 4, our RGP-1.4 and RGP-2 compression methods ranked first and second in mAP0.5 and mAP0.5:0.95 performance among numerous compression techniques, demonstrating the ability of our method to maintain high-precision detection while compressing the model. Additionally, RGP-2 achieved first place in PC (M) and FLOPs (B), highlighting the method’s effective compression capabilities. For FPS, while the advantage of RGP-2 was less pronounced, it still showed an improvement over the baseline model. Therefore, this experiment demonstrates the superiority of our proposed model compression method compared to traditional techniques.

**Table 4:** Comparison results with the mainstream compression method based on YOLOv8n in the GTSDB dataset: the best results are indicated with both bold and underline, while the second-best results are marked with an underline only

Method	mAP0.5	mAP0.5:0.95	PC(M)	FLOPs(B)	FPS
NA (base)	0.920(+0.00%)	0.719(+0.00%)	3.01(+0.00%)	8.1(+0.00%)	95.24(+0.00%)
Ghostnet_050^28^	0.891(-3.15%)	0.681(-5.29%)	2.02(-32.89%)	5.5(-32.10%)	79.37(-16.66%)
Ghostnet_100^28^	0.928(+0.87%)	0.725(+0.83%)	2.77(-7.97%)	6.8(-16.05%)	80.00(-16.00%)
Lcnet_035^29^	0.79(-14.13%)	0.572(-20.45%)	1.97(-34.55%)	5.4(-33.33%)	**116.28(+22.09%)**
Lcnet_050^29^	0.765(-16.85%)	0.556(-22.67%)	2.17(-27.91%)	5.8(-28.40%)	107.53(+12.90%)
Lcnet_075^29^	0.866(-5.87%)	0.632(-12.10%)	2.60(-13.62%)	6.7(-17.28%)	87.72(-7.90%)
Mobilenetv2_035^45^	0.811(-11.85%)	0.6(-16.55%)	2.04(-32.23%)	5.7(-29.63%)	85.47(-10.26%)
Mobilenetv2_050^45^	0.829(-9.89%)	0.622(-13.49%)	2.30(-23.59%)	6.3(-22.22%)	84.03(-11.77%)
Mobilenetv3_small_050^24^	0.827(-10.11%)	0.606(-15.72%)	2.18(-27.57%)	5.4(-33.33%)	80.00(-16.00%)
Mobilenetv3_small_075^24^	0.825(-10.33%)	0.617(-14.19%)	2.61(-13.29%)	5.8(-28.40%)	80.65(-15.32%)
RGP-2 (ours)	0.931(+1.20%)	0.745(+3.62%)	**1.51(-49.83%)**	**4.0(-50.62%)**	101.01(+6.06%)
RGP-1.4 (ours)	**0.937(+1.85%)**	**0.747(+3.89%)**	2.15(-28.57%)	5.8(-28.40%)	92.59(-2.78%)

**Table 5:** Comparison of YOLOv8x Compressed by the RGP Method with Mainstream Detection Models in the GTSDB dataset: the best results are indicated with both bold and underlined, while the second-best results are marked with an underline only

Model	mAP0.5	mAP0.5:0.95	PC(M)	FLOPs(B)	FPS
YOLOv3^41^	0.949	0.784	61.51	154.6	**44.84**
YOLOv5x^42^	0.926	0.725	86.19	203.8	35.21
YOLOv6l^43^	0.929	0.762	59.5	150.5	39.40
YOLOv7x^44^	0.662	0.490	70.8	188.0	29.41
YOLOv8x^8^	0.963	0.792	68.13	257.4	31.35
YOLOx-RGPn (ours)	0.969	0.814	**33.70**	**127.0**	43.86
YOLOx-RGPs (ours)	**0.973**	**0.822**	48.49	183.0	29.33

### Comparison of YOLOv8x compressed by the rigorous gradation pruning method with mainstream detection models

In our previous experiments, we used the smallest version of YOLOv8 as the default baseline. In this section, we will explore the effectiveness of the RGP compression method on the largest version of YOLOv8, specifically YOLOv8x. Similar to the previous experiments, we selected two compressed versions: YOLOx-RGPn and YOLOx-RGPs, which represent models compressed to achieve 2x and 1.4x speed up over the baseline, respectively. Additionally, we compared these compressed models against the largest versions of the currently mainstream YOLO models. The result is shown in Table 5.

In terms of accuracy, YOLOx-RGPs and YOLOx-RGPn ranked first and second, respectively. Notably, in mAP0.5:0.95, YOLOx-RGPs outperformed the third-ranked model by 3.79%. Regarding PC(M) and FLOPs(B), YOLOx-RGPn maintained a leading position, and in FPS, it ranked second, with a difference of less than 1 FPS compared to the first place. Additionally, compared to the baseline model, it achieved a 39.90% improvement in FPS. These results indicate that the RGP method has a significant impact on YOLOv8x, enhancing detection accuracy while reducing computational complexity and parameter count. Moreover, the compressed models demonstrated a clear advantage over other mainstream models.

### Comparison of different speed-up value

Finally, we generated models using different speed up value to intuitively observe the effects of varying degrees of compression. To better understand the impact of different compression, we used the smallest model, YOLOv8n, and the largest model, YOLOv8x, as baselines, and applied different speed up values, resulting in Tables 6 and 7, respectively.

**Table 6:** Comparison results with different speed-up value based on YOLOv8n in the GTSDB dataset: the best results are indicated with both bold and underline, while the second-best results are marked with an underline only

Model	mAP0.5	mAP0.5:0.95	PC(M)	FLOPs(B)	FPS	Speed up
YOLOv8n^8^	0.920(+0.00%)	0.719(+0.00%)	3.01(+0.00%)	8.1(+0.00%)	95.24(+0.00%)	1
YOLO-RGP	**0.937(+1.85%)**	**0.747(+3.89%)**	2.15(-28.57%)	5.8(-28.40%)	92.59(-2.78%)	1.4
	0.931(+1.20%)	0.745(+3.62%)	1.51(-49.83%)	4.0(-50.62%)	101.01(+6.06%)	2
	0.922(+0.22%)	0.726(+0.97%)	0.98(-67.44%)	2.6(-67.9%)	109.89(+15.38%)	3
	0.919(-0.11%)	0.708(-1.53%)	0.58(-80.73%)	1.6(-80.25%)	136.99(+43.84%)	5
	0.873(-5.11%)	0.627(-12.80%)	0.48(-84.05%)	1.3(-83.95%)	129.87(+36.36%)	6
	0.858(-6.74%)	0.649(-9.74%)	0.37(-87.71%)	1.0(-87.65%)	133.33(+39.99%)	8
	0.813(-11.63%)	0.603(-16.13%)	**0.28(-90.70%)**	**0.7(-91.36%)**	**138.89(+45.83%)**	10

**Table 7:** Comparison results with different speed-up value based on YOLOv8x in the GTSDB dataset: the best results are indicated with both bold and underline, while the second-best results are marked with an underline only

Model	mAP0.5	mAP0.5:0.95	PC(M)	FLOPs(B)	FPS	Speed up
YOLOv8x^8^	0.963(+0.00%)	0.792(+0.00%)	68.13(+0.00%)	257.4(+0.00%)	31.35(+0.00%)	1
YOLOx-RGP	**0.973(+1.04%)**	**0.822(+3.79%)**	48.49(-28.83%)	183.0(-28.90%)	29.33(-6.44%)	1.4
	0.969(+0.62%)	0.814(+2.78%)	33.70(-50.54%)	127.0(-50.66%)	43.86(+39.90%)	2
	0.965(+0.21%)	0.808(+2.02%)	22.54(-66.92%)	85.2(-66.90%)	54.35(+73.37%)	3
	0.968(+0.52%)	0.810(+2.27%)	13.42(-80.30%)	50.7(-80.30%)	58.14(+85.45%)	5
	0.970(+0.73%)	0.813(+2.65%)	11.09(-83.72%)	41.8(-83.76%)	59.88(+91.00%)	6
	0.965(+0.21%)	0.800(+1.01%)	8.51(-87.51%)	32.0(-87.57%)	64.10(+104.47%)	8
	0.965(+0.21%)	0.802(+1.26%)	**6.68(-90.20%)**	**25.3(-90.17%)**	**66.67(+112.66%)**	10

As shown in Table 6, the accuracy metrics mAP0.5 and mAP0.5:0.95 tend to decrease with increased compression. However, notably, with a speed-up value of 5, our detection accuracy remains comparable to the baseline model, while PC (M) and FLOPs (B) are reduced by 80.73% and 80.25%, respectively, and real-time performance (FPS) improves by 43.84%. From a speed-up value of 6 onwards, a noticeable decline in accuracy performance occurs, but even at a speed-up of 10, the model still maintains relatively high detection capabilities, with mAP0.5 and mAP0.5:0.95 at 0.813 and 0.603, respectively. At this acceleration level, our lightweight metrics show optimal performance, with PC (M) and FLOPs (B) compressed by 90.70% and 91.36%, respectively, and FPS increased by 45.83%.

As shown in Table 7, compared to the baseline model YOLOv8x, compression consistently leads to performance improvements in mAP0.5 and mAP0.5:0.95. Furthermore, with the increase in speed-up values, the PC (M) and FLOPs (B) decrease significantly. In terms of FPS, the compression effect is particularly notable. At a speed-up value of 10, FPS is improved by 112.66% compared to the baseline.

Regarding the resources required for pruning and retraining, our method is consistent with the training of the baseline model and does not impose excessive demands. Although the addition of the pruning phase inevitably increases the processing time compared to training the baseline model, this added time is relatively short in the context of the entire training process. Moreover, after pruning, the model size is significantly reduced, resulting in a shorter time per training epoch compared to the baseline model. For instance, the training time for the model with a 2x speed up is only 58.33% of the retraining time for the model with a 1.4x speed up. Therefore, if the number of training epochs and the compression ratio are sufficiently large, the overall processing time can be shorter than that of the baseline model.

Additionally, the scalability of the RGP method across the YOLO series of models is also noteworthy. The architectural similarity between different YOLO versions highlights the potential of applying the RGP method to a wide range of YOLO models. With minimal adjustments to the original architecture, the RGP method demonstrates significant flexibility and adaptability.

**Figure 5:**
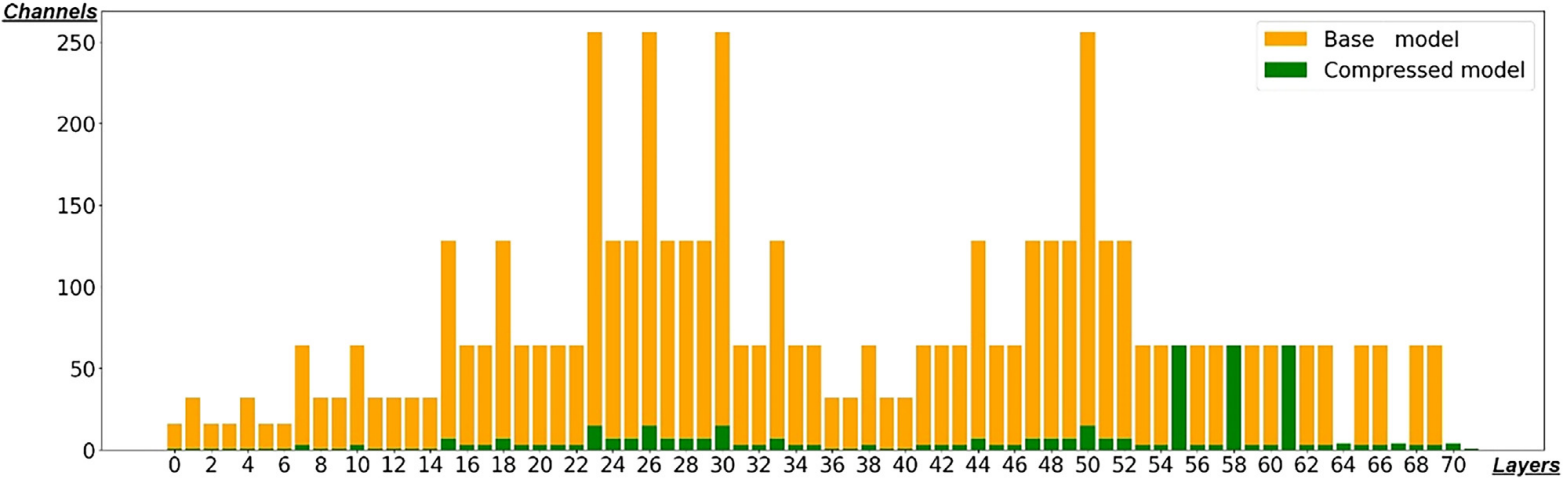
Comparison of compression across different layers in YOLOv8n with a compression rate of 80%

In summary, at various compression levels, our proposed method consistently maintains a reasonable level of detection accuracy. Furthermore, our method offers advantages in processing time and scalability, with continuous improvements in lightweight performance, further validating the effectiveness of our compression approach. See Figure 5 for further real-time detection result, i.e. comparison of compression across different layers in YOLOv8n with a compression rate of 80%.

## Discussion

Model compression in real-time object detection networks presents challenges due to reduced redundancy and diverse architectures. To address these challenges, we proposed the Rigorous Gradation Pruning (RGP) method to compress such network structures. Our RGP approach works by hierarchically pruning filters based on their importance scores, which are calculated using a Desensitized First-Order Taylor method. Furthermore, we reassessed the contribution of each layer in YOLOv8n to the overall output and applied the RGP method, resulting in two versions: YOLO-RGPn and YOLO-RGPs, which achieved compression rates of 50% and 28%, respectively. These two versions proved effective across multiple datasets, including GTSDB, Seaships, and COCO, significantly reducing computational complexity and parameter counts while maintaining detection accuracy.

Additionally, we observed that the compressed versions YOLOx-RGPn and YOLOx-RGPs demonstrated superiority in compressing the network structure of YOLOv8x. Notably, RGP achieved 80% compression on YOLOv8n and 90% on YOLOv8x, while maintaining comparable accuracy. Through a series of experiments, we demonstrated the ability of these compressed models to balance lightweight design with detection performance, validating the effectiveness of our proposed method.

### Limitations of the study

The current RGP method has limitations, particularly a lack of validation through real-world physical experiments, partly due to hardware resource constraints. In the future, we aim to extend our lightweight compression method to newer and larger versions of YOLO and conduct more comprehensive experiments in practical deployment scenarios to further enhance real-time detection models.

## Resource availability

### Lead contact

Requests for further information and resources should be directed to and will be fulfilled by the lead contact, Defu Yang (1002266257@ucsiuniversity.edu.my).

### Materials availability

This study did not generate new unique materials.

### Data and code availability

• This article analyzes existing, publicly available data, accessible at key resources table.
• The code associated with this article can be freely downloaded at the following link: https://github.com/ydf131745/RT-model-compress.
• Any additional information required to reanalyze the data reported in this article is available from the lead contact upon request.

## Acknowledgments

We would like to express our sincere gratitude to the Centre of Excellence for Research, Value Innovation and Entrepreneurship (CERVIE) –UCSI University -Malaysia for supporting this project, with code: REIG-FETBE-2022/022.

## Author contributions

Conceptualization, D.Y. and M.I.S.; methodology, D.Y., Y.Z. and M.I.S.; validation, D.Y., Y.Z. and M.I.S; formal analysis, D.Y.; investigation, D.Y. and Y.Z.; resources, A.A.W., M.I.S., C.C. and B.C.; writing—original draft preparation, D.Y. and M.I.S. ; writing—review and editing, D.Y., W.H.L., A.A.W. and M.I.S.; supervision, M.I.S., C.K.A. and W.H.L. All authors have read and agreed to the published version of the article.

## Declaration of interests

The authors declare no competing interests.

## STAR★Methods

### Key resources table

**Table:** 

REAGENT or RESOURCE	SOURCE	IDENTIFIER
**Deposited data**		

GTSDB	Houben et al.^9^	https://benchmark.ini.rub.de/gtsdb_dataset.html
Seaships	Shao et al.^10^	http://www.lmars.whu.edu.cn/prof_web/shaozhenfeng/datasets/SeaShips%287000%29.zip
MS COCO2017	Lin et al.^11^	https://cocodataset.org/#download

**Software and algorithms**		

YOLOv3	Redmon & Farhadi^41^	https://github.com/ultralytics/yolov3
YOLOv4	Wang et al.^44^	https://github.com/WongKinYiu/PyTorch_YOLOv4
YOLOv5	Jocher et al.^42^	https://github.com/ultralytics/yolov5
YOLOv6	Li et al.^43^	https://github.com/meituan/YOLOv6
YOLOv7	Wang et al.^44^	https://github.com/WongKinYiu/yolov7
YOLOv8	Jocher et al.^8^	https://github.com/DataXujing/YOLOv8
Python	Version 3.9.0	https://www.python.org/downloads/release/python-390/
Pytorch	Version 1.13.0	https://pytorch.org/docs/1.13/

### Experimental model and study participant details

This study utilized three public datasets for experiments, namely GTSDB,^9^ Seaships,^10^ and MS COCO 2017,^11^ all of which encompass both terrestrial and maritime object detection scenarios. The GTSDB targets road signs, containing 900 images with a training-to-testing ratio of 7:3, including categories such as prohibitory, mandatory, danger, and others. Seaships simulate port surveillance of ships using shore-based cameras, featuring 7000 images of 1920 × 1280 pixels with 9221 targets across categories like bulk cargo carrier, container ship, fishing boat, general cargo ship, ore carrier, and passenger ship. The dataset is split into 4900 training images, 700 validation images, and 1400 test images. MS COCO 2017 is a large-scale dataset covering 80 categories of common living objects, with 118287 images in the training set and 5000 in the test set.

The training strategy employed in this experiment uses the same parameters for both initial training and retraining after pruning. For large-scale models or datasets, a batch size of 32 is utilized to accelerate training, whereas a batch size of 4 is employed for smaller models or datasets. The training is conducted over 200 epochs, with 3 epochs dedicated to a warm-up phase to stabilize the learning process and prevent gradient instability during the initial stages. The learning rate is set consistently at 0.01 for both the beginning and end of training. The experiments utilize stochastic gradient descent (SGD) as the optimizer and are implemented in Python 3.9.0.

### Method details

Our proposed RGP pruning method is a structured pruning approach primarily aimed at removing non-important convolution kernels to form a new subnetwork, thereby achieving network compression to ensure minimal loss, as mathematically expressed in Equation 1.

(Equation 1) minFˆ∈FL(Fˆ;(X,Y))=minFˆ∈FL(Fˆ;T),s.t.FˆF≦σ

Here, $X=\left\{x_{1},x_{2},x_{3}\ldots x_{i}\ldots \right\}$ represents the input datasets, $Y=\left\{y_{1},y_{2},y_{3}\ldots y_{i}\ldots \right\}$ the output decisions, and T the collection of inputs and outputs. F is the set of convolutional kernels, $\hat{F}$ is a subnet of F, and σ is the target pruning rate. The objective of pruning is to find an appropriate subnet $\hat{F}$ that minimizes the loss L () while adhering to the pruning rate σ.

Given the vast number of potential subnetworks $\hat{F}$, using an enumeration approach is impractical. Lebedev & Lempitsky, 2016^46^ considered using norm-based convolutional kernel sparsity, as shown in Equation 2:

(Equation 2) $${\left\|F\right\|}_{p}=\sum \sqrt[{p}]{{\left|F_{l}\right|}^{p}}$$

Here, p represents the $\iota_{p}$-norm, commonly employed norms are $\iota_{1}$ and $\iota_{2}$. However, it is important to note that while norm-based sparsity pruning is computationally straightforward and can achieve significant pruning effects, it involves gradually penalizing convolution kernels toward zero. This process can be exceedingly slow, particularly as values approach zero, making it challenging to eliminate them fully. Consequently, this gradual approach to pruning may compromise detection accuracy to some extent.

#### Gradation pruning

Gradation Pruning is chosen over other methods because it provides a more localized and precise pruning strategy. Traditional global pruning strategies, such as those proposed by Khetan & Karnin (2020),^47^ allow for varying degrees of pruning across all layers to enhance pruning efficiency, as illustrated in Figure 2A. However, these strategies do not account for the unique contributions of each layer. In real-time object detection networks, some layers are crucial for maintaining detection accuracy, and a global strategy may inadvertently prune these critical layers, leading to reduced performance. Gradation Pruning, on the other hand, evaluates and prunes layers locally based on their specific contribution to the network’s output, ensuring that essential layers remain intact while gradually reducing the complexity of less crucial layers. This approach balances the need for model compression with the preservation of critical features, making it particularly well-suited for real-time object detection tasks.

To address the concern of selection criteria for crucial layers, we first evaluate each layer’s contribution by measuring its impact on the network’s overall performance. Layers are assessed based on their importance in preserving key features necessary for accurate detection. Layers that are vital for maintaining the accuracy of boundary box sizes, positions, and class predictions are preserved, and no pruning is applied to these crucial layers. This ensures that essential layers remain intact, thus effectively addressing the concern of selection criteria for crucial layers.

Next, within each non-crucial layer, we rank the importance of convolution channels by assigning individual scores based on their relative influence on the output. The pruning process proceeds by systematically removing the convolution kernels with the lowest scores from these non-crucial layers until the condition FˆF≦σ is met. This method allows for a controlled and fine-tuned reduction in model complexity, while ensuring that the overall performance degradation remains minimal. As illustrated in Figure 2B, we employ Gradation Pruning, which is specifically designed to optimize pruning decisions locally for each layer rather than globally.

Additionally, from Figure 2, we can discern that a key aspect of pruning is how each convolution kernel is scored, as this determines which kernel is deemed least important and thus subject to trimming. Previous methods for evaluating the importance scores of convolution kernels typically analyzed the entire kernel as a unit, as exemplified by He et al., 2018,^48^ who used the $\iota_{2}$ norm values to score the entire convolution kernel. However, this approach to scoring based on entire kernels overlooks the impact of individual weights and fails to assess their importance comprehensively. It also requires tailored strategies for different types of kernels, resulting in poor generalizability. To address this, we propose a fine-grained method for assessing the importance of convolution kernels, as shown in Figure 3. Crucially, this involves calculating the importance scores for each weight element and integrating them into a single filter score.

#### Importance assessment based on Desensitized First-Order Taylor

To most straightforwardly express the importance of each element, we set the value of the element to zero and observe the loss in the new network structure compared to the original network structure. Therefore, the importance score of each element can be represented by Equation 3.

(Equation 3) $$S_{n\times m}^{i}={\left(L\left(F;T\right)-L\left(F/F_{n\times m}^{i}=0\right)\right)}^{2}={\left(\frac{\partial L}{\partial F_{n\times m}^{i}}F_{n\times m}^{i}+R_{n\times m}^{i}\right)}^{2}$$

In this context, $S_{n\times m}^{i}$ represents the importance score of the m^th^ element of the n^th^ output channel convolution kernel in the i^th^ layer. We use the Euclidean distance and approximate using a Taylor expansion, where $R_{n\times m}^{i}$ is the higher-order remainder.

To simplify the computation, we have modified the calculation of element importance scores as shown in Equation 4. The purpose of these scores is primarily to rank the relative importance of convolution kernels rather than to compute exact values. Traditionally, the importance score $S_{n\times m}^{i}$ is derived from a Taylor expansion. However, computing higher-order terms $R_{n\times m}^{i}$ is extremely complex, especially in real-time environments, so in our simplification, we first omit this part, forming a first-order Taylor expansion.

Next, we replace the squaring operation with absolute value calculations, making it easier to directly compare the magnitudes of the first-order derivatives without considering their sign. Additionally, because first-order Taylor expansions are highly sensitive to changes in the slope, and convolution kernels in real-time detection models typically exhibit dense gradients that fluctuate sharply, we introduce a desensitization technique.

The core of this desensitization is the use of an exponential function to reduce the sensitivity of the first-order derivatives, ensuring a more balanced comparison between different elements. This transformation mitigates the influence of large gradients, allowing for a fairer comparison of kernel contributions across different layers.

(Equation 4) $$S_{n\times m}^{i}={\left(\frac{\partial L}{\partial F_{n\times m}^{i}}F_{n\times m}^{i}+R_{n\times m}^{i}\right)}^{2}\overset{equal}{\Leftrightarrow }{\left(\frac{\partial L}{\partial F_{n\times m}^{i}}F_{n\times m}^{i}\right)}^{2}\overset{equal}{\Leftrightarrow }\left|\frac{\partial L}{\partial F_{n\times m}^{i}}F_{n\times m}^{i}\right|\overset{equal}{\Leftrightarrow }e^{\frac{\partial L}{\partial F_{n\times m}^{i}}F_{n\times m}^{i}}$$

#### Logical explanation

Taylor Expansion Simplification: Initially, the importance score $S_{n\times m}^{i}$ is derived from the Taylor expansion, which includes the high-order remainder $R_{n\times m}^{i}$. To reduce complexity and improve efficiency, we omit this remainder to forme a first-order Taylor expansion.

Absolute Value Instead of Squaring: We replace the squaring operation with absolute value calculations to make the importance ranking more straightforward, removing the unnecessary influence of gradient signs.

Exponential Function for Desensitization: To address the sensitivity of Taylor expansions to steep gradient changes, we apply an exponential function, which smooths the sensitivity and provides a fairer ranking of kernel contributions across different layers.

The specific convolution kernel scores are shown in Equation 5, which combines the scores of each element.

(Equation 5) $$S_{n}^{i}=S_{n\times 1}^{i}⨁S_{n\times 2}^{i}⨁S_{n\times 3}^{i}...⨁S_{n\times mk^{2}}^{i}$$

Furthermore, to ensure a fairer comparison, we use Equation 6 to perform normalization based on the average value.

(Equation 6) Sni¯=Sni∑Snin

Finally, we obtain a set of scores for the convolution kernels in the i^th^ layer and use Equation 7 to rank them.

(Equation 7) $$S^{i}=Asc.\;Sort\left\{\bar{S_{1}^{i}},\bar{S_{2}^{i}},\bar{S_{3}^{i}}...\bar{S_{n}^{i}}\right\}\overset{\text{Equiv}\;\text{Idx}}{\Rightarrow }F^{i}=Asc.\;Sort\left\{F_{1}^{i},F_{2}^{i},F_{3}^{i}...F_{n}^{i}\right\}$$

The specific process of our proposed RGP model lightweight method is shown in Algorithm 1.

Algorithm 1 **Rigorous global pruning**

**Input:** Full first trained model F.

**Output:** Compressed Model $\hat{F}$.

While FˆF>σ do

 For i in layers:

 For n in input channels:

 $S_{n\times m}^{i}$: score for each element using Equation 4;

 $S_{n}^{i}$: score for each filter using Equation 5;

 Map scores to kernels: $S_{n}^{i}\left[\text{idex}\right]=F_{n}^{i}\left[\text{idex}\right]\text{;}$

 $\bar{S_{n}^{i}}$ = Mean normalization($S_{n}^{i}$).

 $F^{i}=Asc.\;Sort\left\{F_{1}^{i},F_{2}^{i},F_{3}^{i}...F_{n}^{i}\right\}$ acording to value of $S^{i}$.

 If critical layer:

 Pass;

 Else:

 $\hat{F^{i}}$ = remove_filter ($F^{i}$, $F^{i}$ [0]);

 Update $F^{i}$.

 
$\hat{F}=\left\{F^{1},F^{2},F^{3}...F^{i}\ldots \right\}\text{.}$

End While.

**Return:**$\hat{F}$.

#### Implementation of RGP in the real-time detection algorithm YOLOv8

We have selected YOLOv8 as our base model. The network structure of YOLOv8 consists of three main components: the backbone, neck, and head. The backbone is primarily responsible for extracting feature information, the neck processes these features to form semantic information, and finally, the head carries out classification and localization.^8^ The specific implementation process of RGP on the real-time detection algorithm YOLOv8 is depicted in Figure 4. Part A represents the standard real-time model training process, where the YOLOv8 structure is trained using the corresponding dataset to generate a first trained model. Part B utilizes our proposed RGP method for model compression and evaluation of the trained model. Initially, the RGP method prunes non-essential filters to create a new model, the RGP model. It is crucial, however, to first analyze the importance of each layer within the network structure, as layers of critical importance must not be removed. Moreover, it is undeniable that the weights of the RGP model are outdated. If used directly, the detection performance is suboptimal. Therefore, it is necessary to retrain and evaluate using the original dataset to obtain a retrained model. This model is the ideal, streamlined model we recommend.

In order to elucidate Pruning details for each layer in YOLOv8. Figure 5 illustrates that the model achieves relatively uniform compression across all layers, with a very reasonable compression ratio. Additionally, it is important to note that layers 55, 58, 61, 64, 67, 70 and 71 have not been compressed by our method because these are crucial output layers. Layers 55, 58, and 61 are responsible for generating parameters related to bounding boxes, directly impacting the localization of detection boxes, while layers 64, 67, and 70 are primarily involved in classification, affecting the categorization of the detection boxes.

### Quantification and statistical analysis

Regarding evaluation metrics, our assessment will focus on two dimensions: detection accuracy and lightweight. The metrics for detection accuracy are mAP0.5 and mAP0.5:0.95.^49^ The mAP is defined as shown in Equation 8.^50^

(Equation 8) $$mAP=\frac{\sum \sum_{i=1}^{n-1}\left(R_{i+1}-R_{i}\right)\max_{R_{i}\leq R\leq R_{i+1}}P\left(R\right)}{m}$$

In this context, m represents the number of target categories in the dataset, P(R) is the Precision-Recall curve, and n is the number of discrete points on the curve, with R and P being the horizontal and vertical coordinates, respectively. mAP0.5 and mAP0.95 denote the mAP values at IoU thresholds of 0.5 and 0.95, respectively, while mAP0.5:0.95 represents the average mAP value between these two thresholds.

The metrics for evaluating lightweighting include Parameter Count (PC), frames per second (FPS), and floating point operations (FLOPs). The detection time per image includes the cumulative time for preprocessing, inference, and postprocessing. FPS is calculated by dividing 1 s by the detection time taken per image^51^^,^^52^^.^

PC can be calculated using Equation 9,^50^ and FLOPs are divided into FLOPs(con) and FLOPs(full) for convolutional and fully connected layers, respectively, as determined by Equations 10 and 11.^53^

(Equation 9) $$\text{PC}=\left(C_{\text{in}}K^{2}+\delta \left(\text{bias}\right)\right)MC_{out},bias\in \left(0,1\right)$$

(Equation 10) $$\text{FLOPs}\left(\text{con}\right)=\left(2C_{\text{in}}K^{2}-1+\delta \left(\text{bias}\right)\right)SC_{out},bias\in \left(0,1\right)$$

(Equation 11) FLOPs(full)=(2I−1+δ(bias))O,bias∈(0,1)

In this description, $C_{\text{in}}$ and I represent the number of input channels and layers, respectively, while $C_{out}$ and O denote the number of output channels and layers, respectively. K and M correspond to the size and number of convolution kernels, respectively. "bias" refers to the bias term, and S represents the product of the height and width of the output layer.
